# Pattern glare: the effects of contrast and color

**DOI:** 10.3389/fpsyg.2015.01651

**Published:** 2015-10-27

**Authors:** Laura J. Monger, Arnold J. Wilkins, Peter M. Allen

**Affiliations:** ^1^Department of Vision and Hearing Sciences, Anglia Ruskin UniversityCambridge, England; ^2^Department of Psychology, University of EssexColchester, England; ^3^Vision and Eye Research Unit, Anglia Ruskin UniversityCambridge, England

**Keywords:** pattern glare, color, overlays, visual discomfort, reading speed, discomfort threshold

## Abstract

**Aim:** To test a theory of visual stress by investigating the inter-relationships between (1) the threshold contrast/saturation at which individuals first report discomfort when viewing colored gratings of progressively increasing contrast and decreasing saturation; (2) the choice of a colored overlay for reading; (3) any increase in reading speed when the overlay is used.

**Method:** Ninety-five young adults, with normal color vision, reported illusions from square-wave gratings (Pattern Glare Test), chose any colored overlays that improved clarity (Intuitive Color Overlays) and read aloud randomly ordered common words (Wilkins Rate of Reading Test). This was followed by an automated choice of tints for text using various screen colors on a tablet, and a test of discomfort from patterns of progressively increasing contrast and decreasing saturation, using software developed for this study. All participants wore their optimal refractive correction throughout the procedure.

**Results:** Fifty-eight participants chose a colored overlay and reported that it made text easier and more comfortable to read. On average, these individuals had a greater improvement in reading speed with their overlays (*p* = 0.003), a lower contrast threshold at which discomfort from achromatic gratings was first reported (*p* = 0.015), and a tendency to report more pattern glare (*p* = 0.052), compared to the other participants. Participants who chose both a most and least preferred tint for text using the automated procedure reported discomfort from colored gratings at a significantly higher contrast with their most preferred color compared to their least preferred color (*p* = 0.003). The choice of a colored tint was moderately consistent across tests. The most and least preferred colors tended to be complementary.

**Conclusion:** Colored tints that improved reading speed reduced pattern glare both in terms of the illusion susceptibility and in terms of discomfort contrast threshold, supporting a theory of visual stress. An automated test that incorporates colored gratings and a choice of most and least preferred color might better identify individuals whose reading speed improves with colored overlays.

## Introduction

Some individuals experience discomfort and anomalous visual perceptual distortions when they observe patterns of stripes with particular spatial characteristics. Patterns of high contrast having a striped configuration with spatial frequency around 3 cycles/degree, and with stripes of equal width and spacing (duty cycle of ∼50%), tend to produce maximum effect (Wilkins et al., 1984). The sensitivity is greater in patients with migraine or with symptoms of visual stress, and is termed pattern glare (Wilkins et al., 1984; Marcus and Soso, 1989; Harle et al., 2006). The greater sensitivity in migraine is consistent with other evidence of a cortical hyperexcitability (Welch et al., 1990) and it has been proposed that the perceptual distortions reflect the inappropriate spread of excitation (Huang et al., 2011).

Striped patterns are unnatural: their Fourier amplitude spectrum differs markedly from that found in nature. In images from nature the Fourier amplitude spectrum usually approximates 1/f^a^, where *a* is close to 1. On log-log coordinates the spectrum therefore approximates a slope of -1. Juricevic et al. (2010) demonstrated that artificial images having spectra with slopes greater or less than -1 were uncomfortable. Fernandez and Wilkins (2008) showed that discomfort from an image was not dependent simply on the slope but the shape of the Fourier amplitude spectrum. Images with an excess of contrast energy at mid spatial frequencies relative to that expected from 1/f were uncomfortable to view. More recently, Penacchio and Wilkins (2015) considered the Fourier spectrum in two dimensions, and showed that a simple model can explain an average of 27% of the variance in judgments of discomfort from a varied corpus of images. The visual system adapted to natural images, and there are good reasons to suppose that images with 1/f spectra are analyzed by the visual system relatively efficiently (Wilkins and Hibbard, 2014). It is therefore unsurprising that striped patterns, which give rise to an inefficient and less sparse encoding (O’Hare and Hibbard, 2011; Penacchio and Wilkins, 2015) also induce a large haemodynamic response. In general, large haemodynamic responses are associated with discomfort, suggesting that the discomfort is homeostatic and acts to reduce the excessive metabolic demand (Haigh et al., 2015).

The theory of visual stress based on the above findings has been used to explain the benefits of colored tints on reading. Text resembles a striped pattern, partly because of the horizontal rows of words and partly because of the periodic vertical strokes of letters in words such as mum. Both sources of stripes impair reading (Wilkins et al., 2007). Some individuals report visual perceptual distortions of text and these are similar to the illusions of color, shape and motion reported in patterns of stripes (Wilkins and Nimmo-Smith, 1984, 1987). People who experience visual stress often benefit from colored overlays that are placed over passages of text (Jeanes et al., 1997; Wilkins et al., 2001; Hollis and Allen, 2006). The color that provides maximum benefit is specific and individual (Wilkins, 1994). The explanation for this is based on the premise that a hyperexcitability of the visual cortex adversely affects visual processing, and colored filters change the distribution of neural activity so as to reduce the activity in hyperexcitable regions. This hypothesis is difficult to test directly, but is consistent with the neuroimaging evidence that colored filters reduce cortical hyperexcitability (Huang et al., 2011).

There is controversy concerning the use of overlays (Ritchie et al., 2011; Henderson et al., 2012; Uccula et al., 2014), based in part on the various patient selection criteria used. For example, some studies that investigated the effect of colored filters in selected individuals with dyslexia and/or reading difficulties. These individuals are not necessarily those who are likely to benefit from colored filters: a minority of dyslexic individuals have visual stress (Kriss and Evans, 2005).

The following study attempts to link together work on visual discomfort from gratings with that on the effects of colored filters on reading. To date there has been little study of the effects that colored filters might have on patterns of stripes, as opposed to text. Stripes are particularly stressful, for reasons reviewed above, and it is not known whether colored filters are sufficient to reduce the discomfort they evoke. In this study we measured the contrast threshold at which discomfort from stripes was first experienced and we related this threshold to other, more conventional, measures of the benefit from colored filters. Using double-masked methods, three established clinical tests, the Pattern Glare Test, Intuitive Overlays and Wilkins Rate of Reading Test, were followed by an automated examination of color choice and discomfort from patterns of progressively increasing contrast and decreasing saturation, presented on a tablet using software developed for this study. The increasing contrast and decreasing saturation simulated some of the visual effects of those of a colored overlay placed upon a grating as the overlay progressively decreased in saturation, the purpose being to establish a psychophysical threshold for discomfort.

The study seeks an answer to several questions: (1) how can individuals who are likely to benefit from colored filters best be identified?; (2) is the benefit consistent with respect to color? (3) does the benefit generalize to stimuli other than text, including patterns of stripes?

## Materials and Methods

### Participants

The participants were recruited from an undergraduate student population attending Anglia Ruskin University, Cambridge. All procedures conformed to the tenets of the Declaration of Helsinki and were approved by the Anglia Ruskin University Ethics Committee. All participants gave written informed consent after an explanation of the research study.

One hundred young adults (37 male and 63 female), aged between 17 and 31 years (mean = 21.4, *SD* = 2.3) participated. One individual with a history of seizures was excluded.

### Procedure

Four examiners conducted the data collection and were unaware of the findings obtained by the other examiners.

#### Examiner 1

Participants’ monocular and binocular uncorrected vision at distance and near were obtained using a logMAR chart at 6 and 0.4 m. Retinoscopy, monocular refraction and binocular balancing (Modified Humphriss technique) were performed (Humphriss, 1988; Elliott, 2007). Monocular and binocular visual acuities were measured at distance and near. All participants wore their optimal refractive correction throughout the data collection for the following procedures.

The Intuitive Overlays pack (i.O.O Sales Ltd, London, UK) was used to identify participants’ preferences for colored overlays. Each participant was shown colored overlays in the same order, in accordance with the published instructions. Initially the uncovered side of a passage of text was compared to the first color. The least preferred side was changed to the next color, until all colors had been shown. If the participant preferred the uncovered text compared to all nine colors, “no overlay preference” was recorded. If the participant preferred one color they were shown 3 combinations of double overlays in which the second overlay was either the same color or of neighboring chromaticity. At the end each participant was shown their preferred colored overlay/s next to an uncovered page and asked “Do you feel that the overlay makes the text: easier and more comfortable to read, more difficult to read, or is there no difference?”

#### Examiner 2

Each participant was shown the patterned targets from the Pattern Glare Test (i.O.O Sales Ltd, London, UK, 2003), in a random order. The targets were held flat against a wall, at eye level, at a distance of 0.4 m. At this distance, the targets had spatial frequencies of 0.3 cpd, and 2.3 cpd. The 2.3 cpd grating induces glare, and the 0.3 cpd is used simply as a control for any propensity to report marginal visual effects. Participants were asked to look at the fixation spot in the center of each grating. After 5 s, participants were read a list of 15 symptoms in order (red, green, blue, yellow, bending of lines, blurring of lines, shimmering of lines, flickering, fading, shadowy shapes among the lines, pain, discomfort, nausea, dizziness, unease) and asked whether they experienced each symptom. The number of symptoms reported was summed to provide a score for each pattern. The list of symptoms was that used by Allen et al. (2010a) with the addition of ‘fading’.

### Automated Analog of Overlays Selection

**FIGURE 1:**
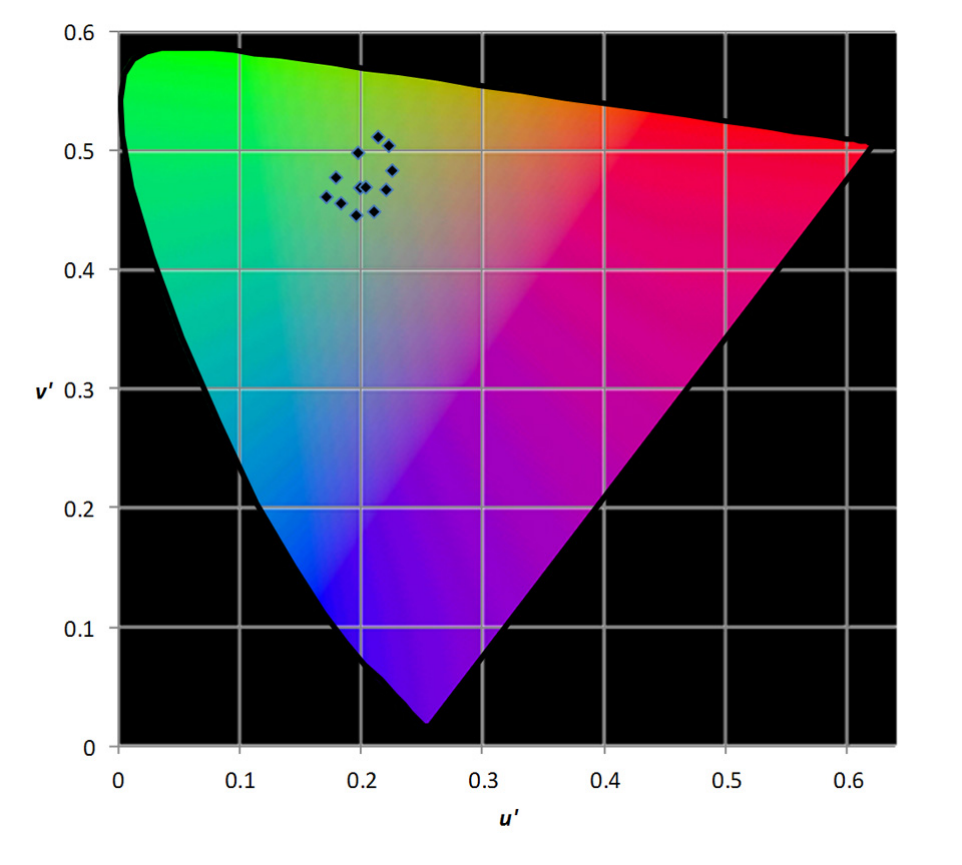
**Chromaticities of colors used in the automated analog of overlays selection**.

Participants then sat in a dark room with an iPad placed at 0.4 m, flat against a wall at eye level. The iPad, screen 198 mm wide and 149 mm high, set at 100% brightness throughout on external power, was used to present two identical passages of text side by side, symmetrically within each half of the screen, generated using PowerPoint and presented using SlideShark 2015 (Brainshark, Inc., Waltham, MA, USA). Each passage consisted of randomly ordered common words (come, see, the, play, look, up, is, cat, not, my, and, dog, for, you, to), 15 words to a line in a different order on each line, in Times 11 pt with 1 pt leading. Each passage, apart from one (maximum contrast text), was tinted by superimposing a rectangular “object” with transparency 50% and one of several different chromaticities. The chromaticities are shown in **Figure 1** and included a white (*u′* = 0.199, *v′* = 0.470), which simply reduced the contrast of the text without changing its chromaticity.

At the outset, the maximum contrast was compared with the reduced contrast. The reduced contrast resulted from the superimposition of the white rectangular object with 50% transparency and this grating was therefore brighter than the remainder (380 cd.m^-2^, compared with 265 cd.m^-2^ (*SD* = 14). After observing the two differently tinted passages, participants touched the screen on the side that was “clearest and most comfortable to read”. The preferred side remained unchanged and the least preferred side changed to the next tint. The tints were presented in the following order: gray, pink, lilac, blue, aqua, green, olive, mustard, orange, peach, rose. The above procedure can be thought of as an automated version of the more conventional clinical examination using overlays. At the end of the procedure, the presentation was repeated but participants chose which color was “most *un*clear and *un*comfortable.”

Finally, color vision was assessed using the Ishihara Test (Kanehara Trading Inc., Tokyo, Japan) and City University Test (Keeler Instruments Inc., USA).

#### Examiner 3

Participants completed the Wilkins Rate of Reading Test (WRRT; i.O.O. Sales Ltd., London, UK) with, without, without and then again with their chosen colored overlays. Each passage was read aloud for 1 min and the number of words read correctly was recorded in words per minute (wpm). For each participant, the average reading speed (wpm) was calculated for the two “with” overlay and the two “without” overlay conditions. The percentage change in reading speed with colored overlays was then calculated for each participant, to allow the effect of colored overlays to be compared between groups of participants.

### Measurement of Discomfort Threshold of Colored Gratings

#### Examiner 4

**FIGURE 2:**
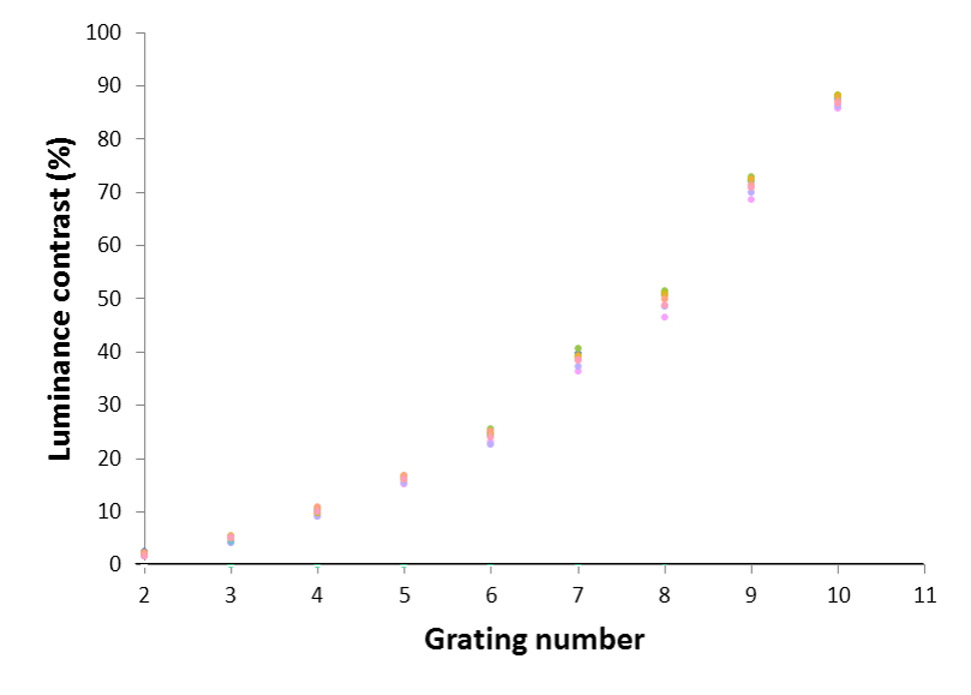
**Luminance contrast of gratings 2–11 for each possible colored overlay choice**.

**FIGURE 3:**
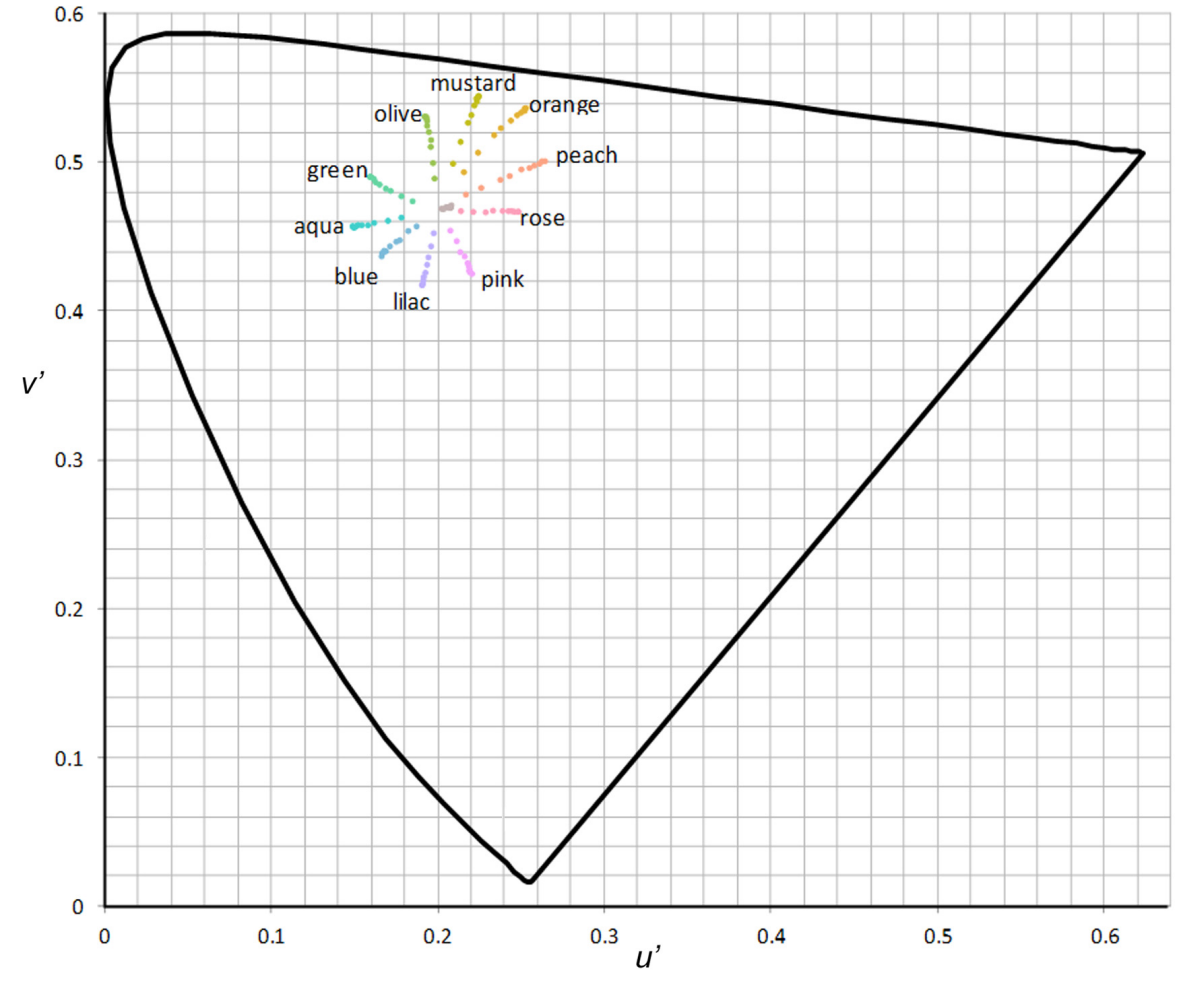
**Chromaticities for the tints at each contrast presentation**.

Participants observed vertical square-wave gratings that progressively increased in contrast. The gratings filled the screen of the iPad at a viewing distance of 0.66 m, at which distance the gratings had a spatial frequency of 2.3 cpd. To measure a threshold for discomfort, participants were asked to look binocularly at the center of the target and report when the pattern first provoked any discomfort. Each grating was covered by a rectangular object which progressively increased in transparency every 3 s as follows: 0, 1, 2, 4, 7, 11, 16, 25, 32, 48, 64%, or until the participant reported discomfort. As a result, the grating increased in contrast as shown in **Figure 2** and its chromaticity progressed toward the chromaticity of white (*u′* = 0.199, *v′* = 0.470) as shown in **Figure 3**. The participants observed three differently tinted gratings in random order. The gratings were (1) gray (all participants); (2) most preferred tint chosen during the automated analog of the overlays selection procedure above (or a randomly chosen colored tint if there was no preferred colored tint); or (3) least preferred colored tint (or randomly chosen colored tint if there was no least preferred colored tint). In other words, individuals who chose the maximum contrast text (380 cd.m^-2^), white tint (380 cd.m^-2^, reduced contrast), or gray tint (266 cd.m^-2^, reduced contrast), as their most or least preferred choice were given a randomly colored tint.

## Results

Five participants had a color vision deficiency. Their results were excluded from the statistical analysis.

### Color and Rate of Reading

The choice of overlays involved successive comparison of nine colored overlays with the white page and any that were marginally preferred to white were subsequently used for comparison in a process of elimination. Unsurprisingly, a large proportion of individuals (86/95) ended the selection procedure having selected one or other of the colors. Of these, 28 reported that the final choice of overlay “made little difference.” There remained 58 participants who reported that the overlays improved the perception of the text. In the mean these individuals read more quickly with their overlay (171 wpm, *SD* = 29.5 with overlay; 159 wpm, *SD* = 28.9 without overlays). For the remaining participants the means were 164 wpm, *SD* = 27.0, with overlay and 160 wpm, *SD* = 29.0, without overlay. Repeated measures analysis of variance with reading rate with and without overlays as factors revealed a highly significant effect of overlay *F*(1,93) = 30.9, *p* < 0.0005 and a highly significant interaction term *F*(1,93) = 8.57, *p* = 0.004, indicating that the effect of overlays was greater among participants who reported that the overlay made the text easier and more comfortable to read.

### Color and Pattern Glare

The individuals who reported greater clarity and comfort with an overlay (*N* = 58) tended to have greater pattern glare; the mean number of symptoms reported with the 2.3 cpd grating was 3.79 (*SD* = 3.44). For the remaining individuals the mean was 2.51 (*SD* = 3.22), (*U* = 822, *z* = 1.95, *p* = 0.052, *r* = 0.2). There were no such differences for the 0.3 cpd (control) grating (*U* = 861, *z* = 1.63, *p* = 0.14).

Thirty-four of the 58 individuals who reported greater clarity and comfort with a colored overlay chose a color (not gray) when examined with the tablet using the automated procedure. Only 10 of the 37 remaining participants chose a color. The preference for a colored tint was therefore moderately consistent across tests, *p* = 0.0032, ϕ = 0.31 (Fisher Exact Probability Test). The chromaticity chosen also showed consistency. This was evaluated by randomization methods. The Pythagorean distance between the chromaticity of each individual’s preferred overlay choice and the preferred chromaticity on the tablet was calculated. The separation for the group on average was 0.065 (*SD* = 0.02). To estimate the separation that might be expected from chance, a participant’s overlay choice was paired with the tablet choice from another participant selected at random. The average separation was calculated. The average for 1000 random combinations was 0.067 (*SD* = 0.00085). The difference was smaller with the original pairing than with the random pairing (*z* = 2.37, *p* = 0.018).

Pattern glare was assessed not only in terms of the symptoms reported at the outset of testing, but also in terms of the threshold contrast of the gratings on the tablet at which discomfort was first reported. The correlation between the number of symptoms reported in response to the 2.3 cpd grating and the threshold contrast with the gray tint was -0.23, *p* = 0.025 (Spearman’s rho). The individuals who reported that a colored overlay made reading more comfortable (*N* = 58) had a lower mean contrast threshold (on the grey tint) of 8.93 (*SD* = 2.01), equivalent to a contrast of 70%, compared with those who did not: 9.92 (*SD* = 1.48), equivalent to 87% (Mann–Whitney *U* = 763, *z* = 2.43, *p* = 0.015, *r* = 0.25). The separation of the groups was larger for the discomfort contrast threshold than for the number of symptoms reported, suggesting that the discomfort threshold provides a more sensitive measure of pattern glare.

The individuals who chose a colored tint (not gray) when examined using the automated procedure on the tablet (*N* = 44) reported more symptoms in response to the 2.3 cpd target than the remaining participants (*N* = 51); 4.50 (*SD* = 3.84) compared to 2.25 (*SD* = 2.58), (Mann–Whitney *U* = 740, *z* = 2.89, *p* = 0.004, *r* = 0.30). Their threshold contrast for discomfort was lower on the gray tint (the grating number averaged 8.73 (*SD* = 2.11), contrast equivalent to 66%) than for those who did not choose a colored tint (9.82, *SD* = 1.48, 85% contrast), (Mann–Whitney *U* = 786, *z* = 2.58, *p* = 0.01, *r* = 0.26).

Twenty-four participants chose both a most and least preferred tint using the automated procedure, and were shown the striped pattern with these color preferences and with gray. Participants reported discomfort at a significantly higher contrast with their most preferred color (9.2, *SD* = 1.9, contrast equivalent to 74%) compared to their least preferred color (8.5, *SD* = 2.4, contrast equivalent to 63%), (Wilcoxon Signed Ranks Test; *z* = 2.95, *p* = 0.003, *r* = 0.60).

Randomization methods were used to determine whether the most preferred and least preferred tints tended to be of complementary color. The CIE UCS chromaticities fell near the circumference of a circle centered on white. This meant that for complementary colors the distance between their chromaticities would be greater than for colors that were not complementary. The Pythagorean distance between the chromaticity of each individual’s preferred tint and least preferred tint was therefore calculated. The separation between the chromaticities of the preferred and least preferred tints for the group on average was 0.043 (*SD* = 0.012). To estimate the separation that might be expected from chance, a participant’s most preferred choice was paired with the least preferred choice from another participant selected at random. The average separation of the chromaticities was then calculated. The random re-pairing was repeated 1000 times and the difference in the chromaticities averaged 0.033 (*SD* = 0.0031). The difference was larger with the original pairing than with the random pairing (*z* = 3.22, *p* = 0.0013).

## Discussion

One would anticipate that individuals susceptible to illusions (pattern glare) would show a greater benefit from colored overlays if susceptibility to illusions in patterns of stripes reflects visual stress, and if colored filters reduce the visual stress, as argued in the introduction. Susceptibility is usually measured in clinical optometric practice simply by counting the number of illusions reported in a grating. This measure was greater in the individuals who reported a benefit from colored overlays under double-masked conditions. A demonstrably more sensitive measure of pattern glare, the threshold contrast at which the pattern induced discomfort, showed that the participants who reported discomfort to gratings with lower contrasts showed a larger increase in reading speed with an overlay. Both measures of pattern glare were consistent and associated with increases in reading speed in the manner predicted by visual stress theory. The above provides a potential answer to the first question posed in the introduction: “How can individuals who are likely to benefit from colored filters best are identified.” It suggests that a more sensitive version of the Pattern Glare test including a measure of discomfort threshold contrast might improve upon existing methods.

Some of the symptoms of visual stress can result from refractive error (Allen et al., 2010b). In this study all participants wore a refractive correction that would have removed any symptoms that might otherwise have been attributable to these refractive errors.

The second question “Is the benefit consistent with respect to color?” is answered by the fact that the participants’ individual choice of color was consistent across tests both with respect to a preference for any color, and the particular chromaticity chosen. It should be noted that the color chosen for a filter is dependent on whether the filter is placed upon a page of text as an overlay, or is worn as spectacles (Lightstone et al., 1999). The optimal color differs in these two contexts, perhaps because of color adaptation. Note that in the present study, the colors were surface colors, but self-luminous in one context and not in the other.

The third question: “Does the benefit generalize to stimuli other than text” is answered at least with regard to gratings. This study has shown that a lower discomfort contrast threshold to gratings is associated with an improvement in reading speed with colored filters. To date the literature on visual discomfort, reviewed in the introduction, has demonstrated a possible mechanism in terms of inefficient neural processing and associated metabolic demand (Wilkins, 2015). The literature on color filters and reading has been largely independent consisting mainly of applied studies (Wilkins, 2003). The present work serves to bring together the theoretical work on discomfort with the practicalities of treatment with colored filters.

In optometric clinical practice visual stress is generally recognized through subjective reports of symptoms and using clinical tests, e.g., the Pattern Glare Test, Intuitive Overlays and Wilkins Rate of Reading Test. It is noteworthy that these clinical findings are now supported with objective evidence of neurological responses associated with this subjective experience. The strength of high-frequency ‘gamma’ oscillations in the primary visual cortex when viewing grating-pattern stimuli was highly correlated with the experience of visual illusions and discomfort (Adjamian et al., 2004). Moreover, imaging studies have revealed an excessive haemodynamic response to stressful patterns in individuals with migraine (Huang et al., 2011) and visual stress (Chouinard et al., 2012). Wilkins and Hibbard (2014) have argued that the discomfort is a homeostatic response, as with other pain.

The current study suggests that an automated test of pattern sensitivity might have two advantages: (1) it can present gratings that increase in contrast and thereby provide a numerical discomfort threshold; (2) it can be quick to choose a most and least preferred color, one assessment corroborating the other. It is possible that those who benefit from colored filters might better be identified using a version of the Pattern Glare test that incorporates plates of increasing contrast, as originally employed by Mulleners et al. (2001). An automated test that incorporates colored stimuli and a choice of most and least preferred color might further differentiate the sensitive individuals.

## Conflict of Interest Statement

Prof. Arnold Wilkins and Prof. Bruce Evans developed the Pattern Glare Test, which is published by i.O.O Sales Ltd. They receive royalties on sales. The other authors declare that the research was conducted in the absence of any commercial or financial relationships that could be construed as a potential conflict of interest.
